# Direct Surface Modification of Polycaprolactone-Based Shape Memory Materials to Introduce Positive Charge Aiming to Enhance Cell Affinity

**DOI:** 10.3390/ma14195797

**Published:** 2021-10-03

**Authors:** Takafumi Zako, Shoko Matsushita, Toru Hoshi, Takao Aoyagi

**Affiliations:** Department of Materials and Applied Chemistry, Graduate School of Science and Engineering, Nihon University, 1-8-14 Kanda-Surugadai, Chiyoda-ku, Tokyo 101-8308, Japan; cstk19022@g.nihon-u.ac.jp (T.Z.); matsushita.shoko@nihon-u.ac.jp (S.M.); hoshi.toru@nihon-u.ac.jp (T.H.)

**Keywords:** shape memory material, polycaprolactone, direct modification, positive charge, cell affinity, mechanobiology

## Abstract

In this study, the introduction of a positive charge on the surface of a shape memory material was investigated to enhance cell affinity. To achieve this, the direct chemical modification of a material surface was proposed. Sheet-type, crosslinked poly(caprolactone-*co*-α-bromo-ɤ-butyrolactone) (poly(CL-*co*-BrBL)) were prepared, and the direct reaction of amino compounds with bromo groups was conducted on the material surface with a positive charge. Branched poly(CL-*co*-BrBL) was prepared, followed by the introduction of methacryloyl groups to each chain end. Using the branched macromonomers, stable and sheet-type materials were derived through UV-light irradiation. Then, the materials were soaked in an amino compound solution to react with the bromo groups under various conditions. Differential scanning calorimetry and surface analysis of the modified materials indicated that 10 vol% of *N, N*-dimethylethylenediamine in *n*-hexane and 1 h soaking time were optimal to maintain the inherent thermal properties. The achievement of increased luminance and a positive zeta potential proved that the direct modification method effectively introduced the positive charge only on the surface, thereby enhancing cell affinity.

## 1. Introduction

Recently, mechanobiology has gained considerable attention in the biomaterial research field [1,2,3,4,5,6], because it allows clarification of the relationship between cell functions and mechanical properties. Moreover, investigation of the surface topography of materials significantly contributes to the field of regenerative medicine. It has been reported that extrinsic factors such as growth environment influences stem cell functions such as differentiation and proliferation in vivo [7]. Previous research has shown that three main factors of scaffold surfaces affect cell functions, namely, chemical, mechanical, and topographical factors [8]. Micro- or nanofabrication technology can effectively realize surface manufacturing. In particular, the effect of topographical features of nanoscale and microscale materials on cell functions such as adhesion, migration, proliferation, and differentiation can be elucidated [9].

Herein, we focused on polycaprolactone (PCL) as a functional material. PCL is useful as a biodegradable and shape memory material. Artificial dura mater has been successfully prepared using a PCL membrane [10,11], and many shape memory materials containing PCL have been reported [12,13,14,15,16]. The use of PCL promotes favorable polymerization initiated by alcoholic compounds and hydroxy group termination. Therefore, molecular design of end groups is possible via the initiation of functional compounds or chemical modification of hydroxy end groups. Previous studies have proven that the molecular design of polycaprolactone facilitated the development of thermo-responsive and shape memory materials without the requirement of additional processes, such as urethane bonding [17,18,19,20]. PCL is a well-known semicrystalline polymer with a melting point of ~60 °C, and its crosslinked materials showed favorable thermo-responsiveness that operated around its melting point. Furthermore, because of the copolymerization with other monomers such as lactide and the introduction of a branched structure in the materials, the crystallinity of the materials was effectively modified to correlate it to their operating temperature [21]. The operating temperature must be adjusted approximately to the body temperature for biomedical applications, and the optimal condition was successfully determined [22].

In addition, surface toughness or patterned topography of film-type PCL can be modified upon changing the temperature. Alternatively, the hydrophilicity and hydrophobicity of the PCL surface do not undergo any change before and after the softening point, that is, a PCL film can be used as the scaffold to understand the influence of PCL on cell functions without considering the temperature-induced changes in hydrophilicity and hydrophobicity [19]. Ebara et al. prepared a shape memory film based on PCL with a patterned surface and used it as a scaffold [20]; they observed that cells were ceded on the patterned scaffold and were lined up along the pattern direction. After heating, the scaffold recovered a flat shape, and the cells randomly migrated. These results suggested that cells can recognize surface morphology and respond to changes in topography. However, PCL has a nonadhesive surface; therefore, it requires a cell-adhesive protein coating such as gelatin or fibronectin. As a result, cells can indirectly interact with the surface via the adhered proteins.

In biomedical field, to improve affinity with a gene or cell for a polymer system, suitable copolymerization was carried out generally [23,24,25,26,27]. Bu et al. reported the surface modification of aliphatic polyesters such as PLA and PCL [28]. By implementing polymer design that improves the affinity of cells to the material surface, the introduction of a positive charge on the PCL surface allowed the enhancement of cell affinity. This could be attributed to the negative charge of the cell surface due to the presence of sialic acids at the sugar chain ends. To achieve this, crosslinked PCL films with a positive charge on their surfaces have been designed and prepared [29]. The enhancement of cell adhesion without any protein precoating was confirmed; however, controlling the positive charge density of PCL films was not easy. To simplify it, α-bromo-ɤ-butyrolactone (BrBL), which has a reactive Br group, can be used to introduce a positive charge. BrBL copolymerizes with ε-caprolactone to afford copolymer poly(CL-*co*-BrBL) with Br groups of the polymer main chains [30]. Thus, a positive charge can be introduced by using a simple reaction with amino compounds and Br groups.

In this study, we intended to introduce a positive charge only to the surface of the film by soaking the film in an amine solution. Such direct surface modification would be advantageous because it can be functionalized without influencing bulk properties. Moreover, the film is expected to maintain positive charge density before and after expansionary deformation. To achieve the research objectives, the actual studies consist of (a) copolymer synthesis and its characterization, (b) film preparation and its shape memory property, (c) investigation of optimal condition for introduction of amine groups and confirmation of maintenance of bulk properties, (d) characterization of the positively-charged surface, and (e) preliminary test for cell affinity.

## 2. Materials and Methods

### 2.1. Materials

ε-Caprolactone 1,4-butanediol and pentaerythritol as initiators were obtained from the Tokyo Chemical Industry (TCI), Tokyo, Japan. α-Bromo-ɤ-butyrolactone (BrBL) was commercially available from Wako Pure Chemicals, Tokyo, Japan. Tin dioctanoate as a polymerization catalyst, methacryloyl chloride *N, N*-dimethylethylenediamine (DMEDA), and 2-hydroxy-4-(2-hydroxyethoxy)-2-methylpropiophenone were purchased from TCI (Tokyo, Japan) and used as received. Solvents such as tetrahydrofuran, chloroform, ethyl acetate, and *n*-hexane, which were used for the reaction or polymer purification, were reagent grade and used as received. Anionic dye 2′,4′,5′,7′-tetrabromofluorescein disodium salt (Acid Red 87) were acquired from TCI and were used in the characterization with the amino compound and for positive charge introduction. HeLa cells (JCRB9004) were purchased from JCRB cell bank of National Institutes of Biomedical Innovation, Health, and Nutrition, Osaka, Japan.

### 2.2. Characterization

To calculate the monomer content in the copolymer, ^1^H-NMR spectra were recorded using a JEOL RESONANCE spectrometer (JNM-ECP500; Tokyo, Japan) operated at 400 MHz. Deuterated chloroform (CDCl_3_) was used as a solvent, and chemical shifts of the peaks were recorded with respect to tetramethylsilane (TMS). The thermal properties of the prepared materials were investigated through differential scanning calorimetry (DSC, DSC6100, Seiko Instruments Inc., Tokyo, Japan). The heating rate was 10 °C/min, and the data from the second scan were adopted. The photoluminescence of the adsorbed fluorescent probes was evaluated through a fluorescence spectrophotometer (Scope.A1, ZEISS Research Microscopy Solutions, GmbH, Jena, Germany) to estimate the positive charge. Moreover, the surface characteristics were studied on the basis of the ATR-IR spectra recorded by the Spectrum On system (PerkinElmer, Billerica, MA, USA). Zeta potential was measured using a zeta potential analyzer (ELS-2000ZS, Otsuka Electronics, Osaka, Japan).

### 2.3. Preparation of Two-Branched and Four-Branched Polycaprolactone-co-α-bromo-ɤ-butyrolactone (2bPolyCL-co-BrBL-OH and 4bPolyCL-co-BrBL-OH, Respectively)

**Figure 1:**
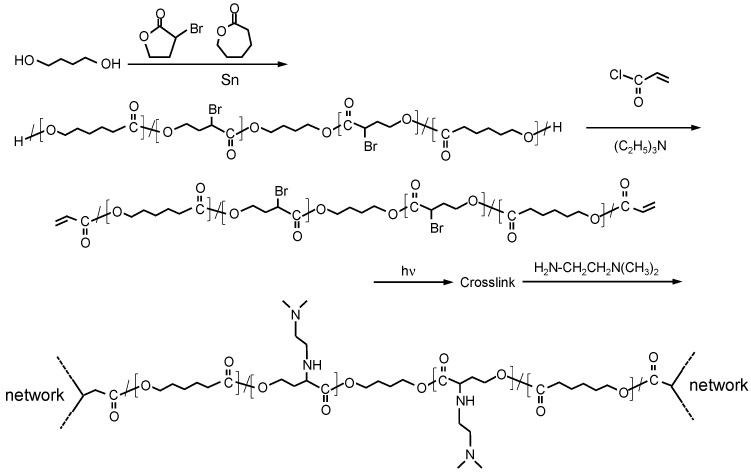
Preparative schemes of branched PolyCL-co-BrBL-OH, its macromonomer, and amine-modified crosslinked materials.

Preparative scheme of starting branched copolymer, macromonomers, and amine-modification reaction is shown in Figure 1.

The branched PolyCL-*co*-BrBL-OH was prepared through the conventional ring-opening polymerization of CL and BrBL, initiated using 1,4-butandiol or pentaerythritol. For preparing 2bPolyCL-*co*-BrBL-OH, a few drops of stannous octanoate were added to a flask containing dried 1,4-butandiol with 20.0 g CL and 7.2 g BrBL. The reaction mixture was stirred under dry argon atmosphere at 110 °C for 5 h. After cooling, the resulting viscous liquid was diluted with chloroform and poured into a mixture of ethyl acetate and *n*-hexane (1:4 v/v). The desired 2bPolyCL-*co*-BrBL-OH was obtained as a white powder (yield 75.6%). ^1^H-NMR δ (CDCl_3_, ppm): 1.38 (m, –CO–CH(Br)CH_2_CH_2_O-/-CO–CH_2_CH_2_C*H*_2_CH_2_CH_2_O-), 1.65 (m, –CO–CH(Br)CH_2_CH_2_O-/-CO–CH_2_C*H*_2_CH_2_C*H*_2_CH_2_O-), 2.31 (m, –CO–CH(Br)CH_2_CH_2_O-/-CO–C*H*_2_CH_2_CH_2_CH_2_CH_2_O-), 2.41 (m, –CO–CH(Br)C*H*_2_CH_2_O-/-CO–CH_2_CH_2_*C*H_2_CH_2_CH_2_O-), 3.65 (m, –CO–CH(Br)CH_2_CH_2_O-/-CO–CH_2_CH_2_CH_2_CH_2_CH_2_O-C*H*_2_OH (chain end)), and 4.06 (m, –CO–CH(Br)CH_2_CH_2_O-/-CO–CH_2_CH_2_CH_2_CH_2_C*H*_2_O-), 4.23 (m, –CO–CH(Br)CH_2_C*H*_2_O-/-CO–CH_2_CH_2_CH_2_CH_2_CH_2_O-), and 4.34 (m, –CO–C*H*(Br)CH_2_CH_2_O-/-CO–CH_2_CH_2_CH_2_CH_2_CH_2_O-). Next, 7.0 mL of triethylamine and 7.0 mL of methacryloyl chloride were added to a chloroform solution containing 20.0 g of 2bPolyCL-*co*-BrBL-OH prepared using the abovementioned procedure and stirred for 24 h. The reaction mixture was subsequently purified with a salt through liquid separation and poured into a mixture of ethyl acetate and *n*-hexane to purify 2bPolyCL-*co*-BrBL-MA. The desired 2bPolyCL-*co*-BrBL-MA was obtained as a light-yellow-colored powder (yield 92.4%). ^1^H-MR δ (CDCl_3_, p.p.m.): 1.38 (m, –CO–CH(Br)CH_2_CH_2_O-/-CO–CH_2_CH_2_C*H*_2_CH_2_CH_2_O-CO-C(CH_3_)=CH_2_, 1.64 (m, –CO–CH(Br)CH_2_CH_2_O-/-CO–CH_2_C*H*_2_CH_2_C*H*_2_CH_2_O-CO-C(CH_3_)=CH_2_), 1.94 (m, –CO–CH(Br)CH_2_CH_2_O-/-CO–CH_2_CH_2_CH_2_CH_2_CH_2_O-CO-C(C*H*_3_)=CH_2_), 2.31 (m, –CO–CH(Br)CH_2_CH_2_O-/-CO–C*H*_2_CH_2_CH_2_CH_2_CH_2_O-CO-C(CH_3_)=CH_2_), 2.48 (m, –CO–CH(Br)C*H*_2_CH_2_O-/-CO–CH_2_CH_2_*C*H_2_CH_2_CH_2_O-CO-C(CH_3_)=CH_2_), and 4.05 (m, –CO–CH(Br)CH_2_CH_2_O-/-CO–CH_2_CH_2_CH_2_CH_2_C*H*_2_O-CO-C(CH_3_)=CH_2_), 4.28(m, –CO–CH(Br)CH_2_C*H*_2_O-/-CO–CH_2_CH_2_CH_2_CH_2_CH_2_O-CO-C(CH_3_)=CH_2_), 4.38 (m, –CO–C*H*(Br)CH_2_CH_2_O-/-CO–CH_2_CH_2_CH_2_CH_2_CH_2_O-CO-C(CH_3_)=CH_2_), 5.55, and 6.09 (m, –CO–CH(Br)CH_2_CH_2_O-/-CO–CH_2_CH_2_CH_2_CH_2_CH_2_O-CO-C(CH_3_)=C*H*_2_). The same procedure was adopted to prepare the 4-branched PolyCL-*co*-BrBL-MA using 4bPolyCL-*co*-BrBL-OH with pentaerythritol as an initiator.

### 2.4. Preparation of Shape Memory Film with Two-Branched and Four-Branched PolyCL-co-BrBL-MA (2bPolyCL-co-BrBL-MA and 4bPolyCL-co-BrBL-MA, Respectively)

One gram of the mixture of the two-branched and four-branched PolyCL-*co*-BrBL-MA was dissolved in 1.5 mL THF containing a photosensitizer. This solution was poured into a 5.0 × 5.0 × 0.1 cm mold and quickly placed between a PET sheet and a glass plate. A light-triggered crosslinking reaction occurred upon irradiation of each side of the plate for 15 min by using a high-pressure mercury lamp (UVL-100HA (100 W), Rikokagaku Co., Tokyo, Japan). The lamp has some line spectra at 254, 313, 365 (main), 405, and 436 nm and was used without any filter. The obtained PolyCL-*co*-BrBL film was swollen in acetone for 1 d and then dried in a vacuum oven. Eventually, light-yellow-colored sheets were obtained.

### 2.5. Direct Surface Modification of PolyCL-co-BrBL Film Using Amino Compound Solution

**Figure 2:**
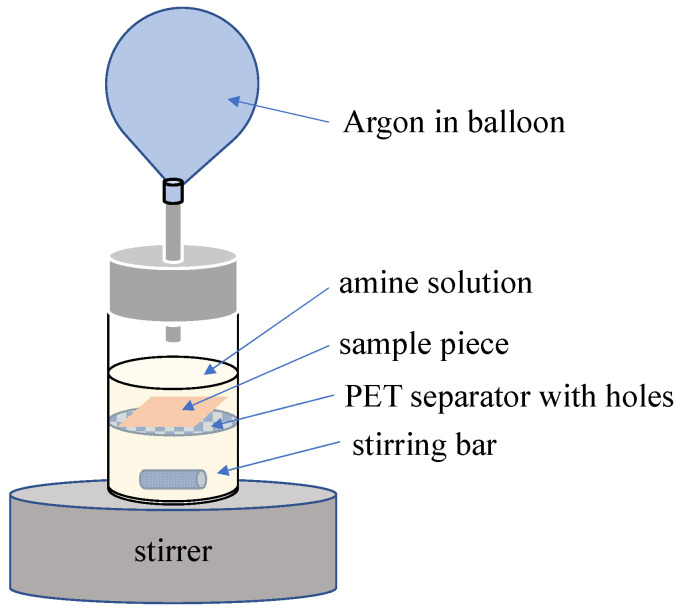
Amine modification procedure. The sample piece was separated by the PET film with holes to prevent collision with the stirring bar.

Representative method to modify amine onto the materials surface is shown in Figure 2. The PolyCL-*co*-BrBL film, having dimensions of 1.0 × 3.0 × 0.1 cm, was cut and soaked into DMEDA solution of *n*-hexane. The apparatus is shown in Figure 2. The sample piece was put on the PET film with many holes fixed in the solution so as not to collide with the stirring bar. The solution was stirred under dry argon atmosphere at 25 °C. Then, solution was changed to 50 mL of pure *n*-hexane and stirred for 10 min to wash out the remaining amine. The operation was repeated twice to remove the amine thoroughly. The obtained cationic film was dried in a vacuum oven. Eventually, white-colored films were obtained. In this study, we investigated the effects of the amino compound solution concentration and reaction time on the film surface properties.

### 2.6. Contact Angle Measurement

The contact angles were measured to estimate the introduction of amino groups onto the surface. Such group was expected to show more hydrophilic property. We put a 2 μL water droplet on the sample and measured the contact angles using goniometer (No.20424, Erma CO., Tokyo, Japan) and calculated by a half-angle method. The measurement was repeated 3 times, and the value was averaged from 6 points of the one sample.

### 2.7. Preparation of Stretched Modified PolyCL-co-BrBL Film

The shape memory of the modified films was investigated using a temperature-controlled oven. At 50 °C, the specimens of the prepared films with a size of 1.5 cm × 5 cm × 0.5mm were fixed at the end using a clip in the oven and left for 30 min. Then, a weight of 250 g was hung on the other end of the specimens; this setup was maintained until the films cooled down to room temperature.

### 2.8. Cell Adhesion onto Modified PolyCL-co-BrBL Film

Before starting the cell culture, the modified PolyCL-co-BrBL film was sterilized using a 70% ethanol aqueous solution. The specimen was stuck on the glass plate, and the small silicone chamber was placed on the plate. The same three samples were provided to the experiment. HeLa cells were seeded at a density of 7.0 × 10^4^/cm^2^ cells or were put on a sterilized material surface and cultured in a medium at 37 °C for 16.5 h. The HeLa cells on the surfaces were fixed with 4% paraformaldehyde for 10 min and permeabilized with 0.1% Tween 20/PBS for 5 min at room temperature. To visualize the nucleus, the cells were treated with Hoechst 33,342 for 1 h. The cell morphology was imaged using a fluorescence microscope (Olympus IX70, Tokyo, Japan).

## 3. Results and Discussion

### 3.1. Preparation of Crosslinkable Poly(CL-co-BrBL) as a Starting Material

**Figure 3:**
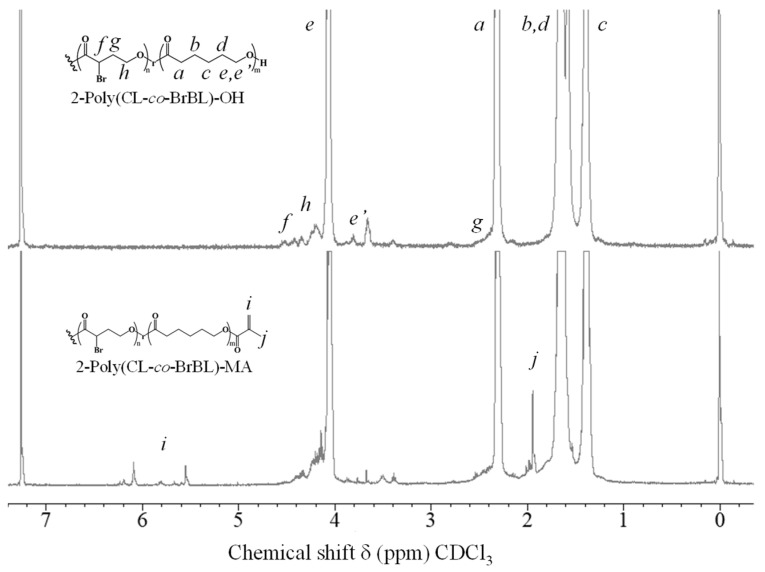
^1^H-NMR spectra of 2-poly(CL-co-BrBL)-OH as a precursor and 2-poly(CL-co-BrBL)-MA as a macromonomer. The spectra (d, ppm) were recorded with TMS as standard in CDCl_3_.

According to our previous report, crosslinkable, branched, cationized PCL-based macromonomers were successfully prepared, and materials with positively charged surfaces were derived from macromonomers [29]. The quaternized cationic group was introduced by the reaction with bromoacetyl bromide substituted to hydroxy groups, followed by the reaction with N, N-diethylaminomethyl methacrylate. In these materials, the cationic groups localized around crosslinked points because the quaternized cationic group was derived from the reaction between N, N-dimethylaminoethyl methacrylate and bromoacetyl groups at the chain end. Therefore, in this study, we intended to prepare other types of positively charged materials wherein the cationic groups would be distributed evenly on the material surface. To achieve this, the copolymers containing CL and BrBL were synthesized by a previously reported method [30] because the cationic group introduction was expected to be easily achieved after the reaction with bromo groups. Figure 3 shows the ^1^H-NMR spectra of the starting poly(CL-co-BrBL) and its macromonomer. According to the figure, specific peaks derived from CL and BrBL (upper spectrum in Figure 3) and additional peaks based on the methacryl groups in poly(CL-co-BrBL) are recognized. These results suggest the successful preparation of the objective materials.

### 3.2. Preparation of Brominated PCL-Based Crosslinked Materials

**Figure 4:**
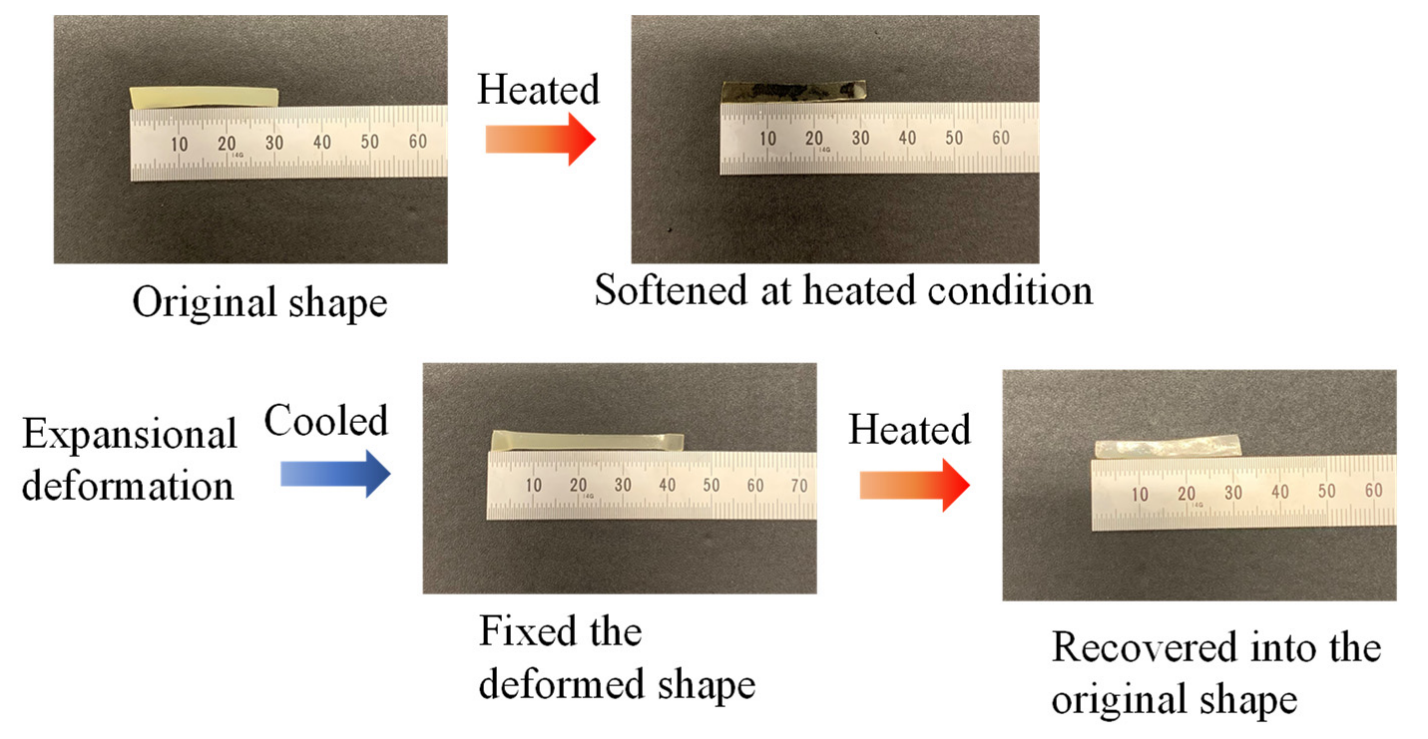
Shape memory of the crosslinked 2-poly(CL-co-BrBL)-MA. The memorized, expansional deformed shape recovered into the original shape by heating over the softening point.

Table 1 summarizes the preparation results and thermal properties of the branched macromonomers composed of copolymers CL and BrBL. The composition ratios of BrBL were calculated from the ^1^H-NMR spectra, and the thermal properties were studied through DSC. The results indicated that the composition of BrBL in the copolymers was one-tenth that of the feeding ratio, which could be due to the relatively stable structure of the ring-opened BrBL. The literature that dealt with copolymerization of CL and BrBL showed that a larger ratio was obtained in the same condition [30]. The reason for the resulting lower ratio of introduced BrBL was unclear; however, larger introduced BrBL ratio was obtained by increased molar ratio of BrBL for copolymerization, as indicated in Table 1. The four-branched CL-co-BrBL did not show a peak in the DSC chart, indicating that the melting point was beyond the temperature range. Its lower melting point was due to the presence of the branched structure and the disrupted copolymerization owing to polymer crystal formation. Therefore, we considered that the presence of the four-branched structure may significantly decrease the melting point; thus, direct surface modification can be studied only in the presence of a two-branched structure. Figure 4 shows shape memory of (2-Poly(CL-co-BrBL) film) using its strip sample, and it memorized expansional deformation and recovered into original shape by heating over the softening point. Its recovery ratio (R%) was estimated by size measurement method [31]. The value was almost 100%, and it indicated this sample did keep its bulk property, even in the deformation process.

### 3.3. Influence on Bulk Properties Based on Reaction Conditions

**Figure 5:**
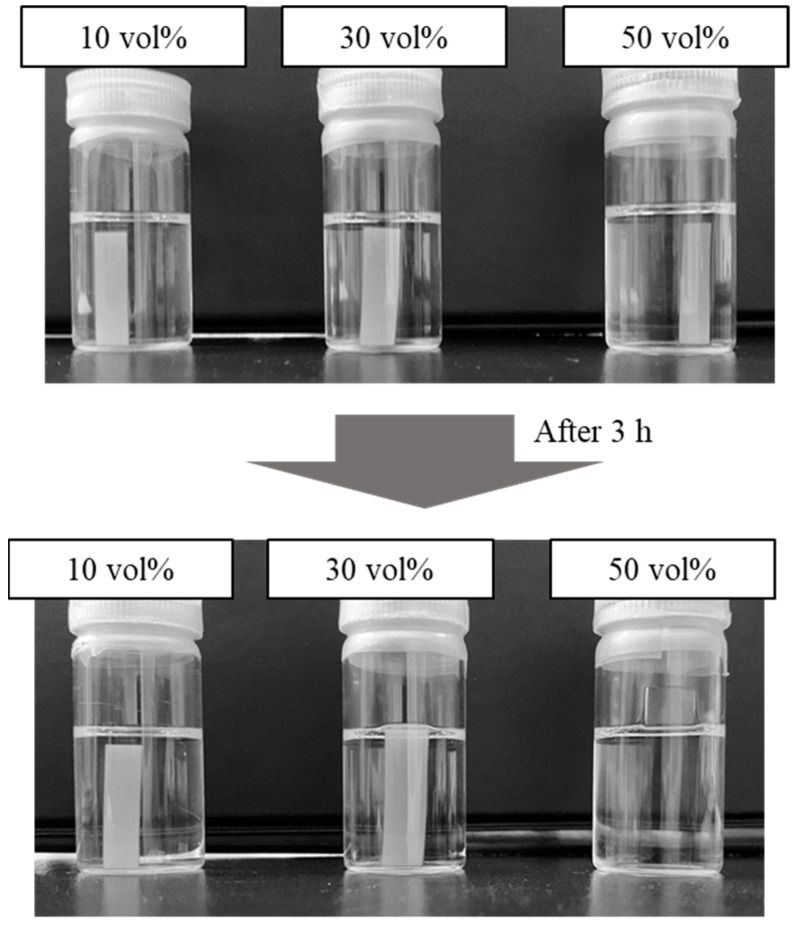
Photos of shape memory materials in reaction solution containing DMEDA with varied concentrations. The percentage indicated DMAEA concentrations.

**Figure 6:**
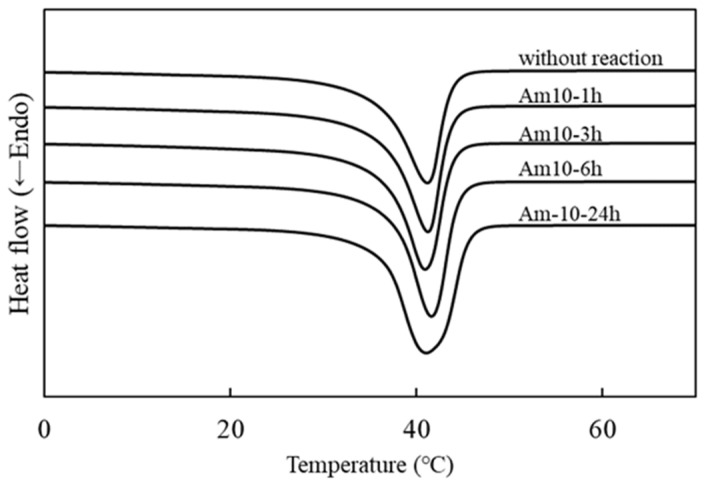
DSC charts of the samples before and after reaction with DMEDA. Symbols of samples are indicated in Table 2.

To obtain surface-modified shape memory materials, the surface was modified to acquire a positive charge, and brominated materials were soaked in the DMEDA solution of n-hexane for 1, 3, 6, and 12 h. For the preliminary experiment, benzylamine (BA) and trimethylamine (TMA) were tested to investigate suitable amine structure. BA could react with Br groups, but TMA could not. Steric hinderance of TMA might obstruct the reaction. From that result, DMEDA that contained both primary and tertiary amine was selected for the reaction. Furthermore, some organic solvents such as swellable tetrahydrofuran and nonswellable methanol were tested to prepare the amine solution. The swelling of the materials allowed the reaction at the surface and in the bulk phase as well as changed the thermal properties. The use of tetrahydrofuran causes swelling of the materials and changed the softening point and the value of enthalpy change. Alternatively, methanol did not cause material swelling; however, methanolysis unexpectedly occurred, and the material surface was eroded [32]. Therefore, we selected n-hexane as the nonswellable and nonerodible solvent for direct modification on the surface. In this experiment, solutions having 10, 30, and 50 vol% of DMEDA in n-hexane were used. Figure 5 shows an image of the soaked samples in the reaction solutions. As seen in the figure, the sample size became larger in 30 vol% of amine compound and swelled in 50 vol%. For example, the sample height become almost 1.3-fold larger in 30vol% condition and 1.8-fold in 50 vol% larger. This result suggests that the reaction proceeded in the bulk phase; resultantly, change of the softening point was concerned. That is to say, it was observed that high amine concentration enhanced material swelling; at low concentration, amino compounds could effectively react with the material surface. Thus, at low concentration condition with 10% of DMEDA, the reaction was conducted. Figure 6 shows the DSC charts of the samples with different reaction times of 1, 3, 6, and 12 h, and the results are summarized in Table 2. The T_m_ and ΔH values of each sample were almost equal because the unexpectable reaction in the bulk phase could be avoided at low amine concentration. However, the peak shape became broader with increasing reaction time, and the result suggested short reaction time was preferable to maintain inherent thermal property.

### 3.4. Evaluation of Surface Modification of Poly(CL-co-BrBL)-Based Materials

**Figure 7:**
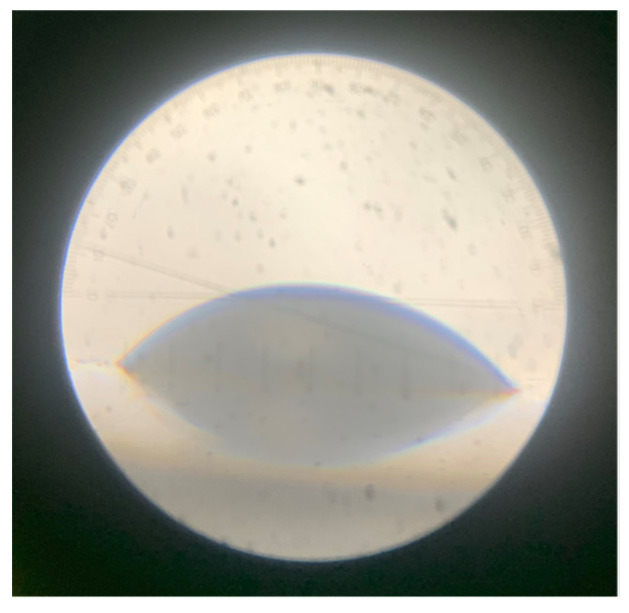
Actual image water droplet on the amie-modified surface at contact measurement.

**Figure 8:**
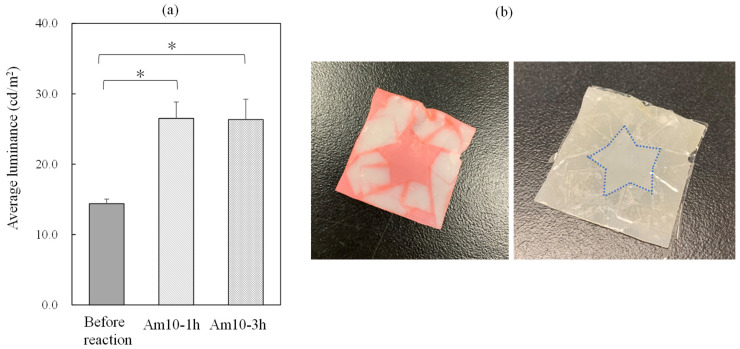
Anionic dye adsorption onto the sample surface before and after reaction with DMEDA. (**a**) Estimation by calculated average luminance. *n* = 3. (**b**) The dye molecule interaction with selected adsorption area controlled by masking. White area was masked using adhesive tape. *, the results are statistically significant, *p* < 0.01.

**Figure 9:**
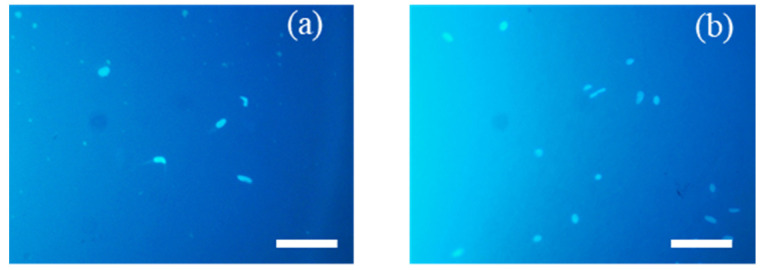
Microscopic image of adhered HeLa cells on the surface (**a**) before and (**b**) after the reaction with DMEDA. Scaling bars indicate 100 μm.

To confirm surface modification, contact angle measurements were conducted. Table 3 shows the contact angles of the water droplets on the material surfaces. Its actual image is shown in Figure 7. As expected, reaction with the amine compound led to enhanced hydrophilicity, and certainly, the more charged surface meant the greater interaction with water and showed lower contact angle value. In a previous study, the anionic dye adsorption test was concluded to be easy and useful in evaluating the positive charge of a material surface [29]. Therefore, the same experiment was conducted, and the observed luminescence is indicated in Figure 8a. Interestingly, the samples Am10-1 h and -3 h showed the larger intensities compared with unreacted one. These results mean the surface turned into positive and promoted the interaction with anionic compound. Furthermore, we tried to make a pattern of positive charge by masking the method preliminarily. In Figure 8b, only the positively charged surface was obtained at the area without masking and it was promising to design the patterning of the materials surface. Zeta potentials of both samples before and after reaction (Am10-1 h was used) were measured to be −5.6 ± 0.6 and 20.1 ± 2.1 mV. This values also indicated successful reaction with DMEDA. Finally, cell affinity to the prepared materials was investigated preliminarily. Figure 9 shows the images of the HeLa cells adhered to the surfaces of the starting and amine-modified materials. The number of cells adhered to the modified samples was almost two times larger than that to the starting material, that calculated from the pictures, which indicates that the materials in this study could improve the cell affinity. Now, we have been studying quantification. Consequently, these materials can have practical applications in mechanobiology, specifically for enhancing cell–material interaction.

## 4. Conclusions

To impart cell affinity to the shape memory material surface while maintaining the material’s thermal property, direct modification was studied. The macromonomer derived from copolymer poly(CL-co-BrBL)-MA was used as the starting material to prepare the shape memory material. After the crosslinking reaction, stable materials were obtained and were subjected to direct surface modification. The use of a nonswellable solvent and optimization of amine concentration and reaction time facilitated the reaction only on the surface without preventing unexpected reactions in the bulk phase. The results of DSC, contact angle, and zeta potential measurement, and anionic dye adsorption tests suggested that a successful reaction occurred and positively charged material surface was formed. Moreover, the cell adhesion study proved that the material was able to maintain cell affinity. Consequently, the direct modification investigated in this study can be considered for the introduction of cell affinity, and the as-prepared materials can be applied in mechanobiology to explore cell–material interactions.

## Figures and Tables

**Table 1:** Preparation of branched copolymers and macromonomers comprising CL and BrBL.

Sample	Feed Ratio [BrBL/CL]	Composition Ratio [BrBL/CL]	Yield [%]	Introduction Ratio of MA [%]
2-Poly(CL-co-BrBL)-OH	0.25	0.04	94.1	-
2-Poly(CL-co-BrBL)-OH	0.50	0.07	34.1	-
4-Poly(CL-co-BrBL)-OH	0.13	0.02	40.5	-
4-Poly(CL-co-BrBL)-OH *	0.25	-	-	-
2-Poly(CL-co-BrBL)-MA	0.25	0.04	64.3	82.6
4-Poly(CL-co-BrBL)-MA	0.13	0.02	34.4	72.3

* This sample was not used for macromonomer preparation.

**Table 2:** Thermal properties of the materials from different reaction times.

Sample	*T*m (°C)	D*H*m (mJ/mg)
Before reaction	41.2	49.7
Am10-1 h	41.3	50.3
Am10-1 h	40.9	49.5
Am10-1 h	41.4	53.0
Am10-1 h	41.1	57.3

**Table 3:** Contact angle measurements.

Sample	Reaction Time (h)	Contact Angle * (°)
Before reaction	-	57.9 ± 6.0
Am10-1 h	1	42.4 ± 5.5
Am10-3 h	3	44.9 ± 9.0

* The values were obtained by average of n = 18.

## Data Availability

Data is contained with the article.
